# Elucidating the Biomechanics of Leukocyte Transendothelial Migration by Quantitative Imaging

**DOI:** 10.3389/fcell.2021.635263

**Published:** 2021-03-29

**Authors:** Amy B. Schwartz, Obed A. Campos, Ernesto Criado-Hidalgo, Shu Chien, Juan C. del Álamo, Juan C. Lasheras, Yi-Ting Yeh

**Affiliations:** ^1^Department of Mechanical and Aerospace Engineering, University of California, San Diego, La Jolla, CA, United States; ^2^Department of Bioengineering, University of California, San Diego, La Jolla, CA, United States; ^3^Institute of Engineering in Medicine, University of California, San Diego, La Jolla, CA, United States; ^4^Department of Mechanical Engineering, University of Washington, Seattle, WA, United States; ^5^Center for Cardiovascular Biology, University of Washington, Seattle, WA, United States

**Keywords:** leukocyte, vascular endothelial cell, transednothelial migration, biomechanics, force microscopy

## Abstract

Leukocyte transendothelial migration is crucial for innate immunity and inflammation. Upon tissue damage or infection, leukocytes exit blood vessels by adhering to and probing vascular endothelial cells (VECs), breaching endothelial cell-cell junctions, and transmigrating across the endothelium. Transendothelial migration is a critical rate-limiting step in this process. Thus, leukocytes must quickly identify the most efficient route through VEC monolayers to facilitate a prompt innate immune response. Biomechanics play a decisive role in transendothelial migration, which involves intimate physical contact and force transmission between the leukocytes and the VECs. While quantifying these forces is still challenging, recent advances in imaging, microfabrication, and computation now make it possible to study how cellular forces regulate VEC monolayer integrity, enable efficient pathfinding, and drive leukocyte transmigration. Here we review these recent advances, paying particular attention to leukocyte adhesion to the VEC monolayer, leukocyte probing of endothelial barrier gaps, and transmigration itself. To offer a practical perspective, we will discuss the current views on how biomechanics govern these processes and the force microscopy technologies that have enabled their quantitative analysis, thus contributing to an improved understanding of leukocyte migration in inflammatory diseases.

## Introduction

Leukocytes encompass a diverse group of white blood cells in the immune system, including lymphocytes, monocytes, dendritic cells, and neutrophils, which exhibit a versatile and broad range of migratory abilities. Leukocyte migration from the bloodstream to sites of injury or infection is a primary component of the innate immune and inflammatory responses. Functioning as first responders, leukocytes can efficiently overcome biophysical barriers in their response to pro-inflammatory stimuli, including the vascular wall and dense three-dimensional (3-D) extravascular spaces. This efficient pathfinding is essential for leukocyte trafficking and provides potential therapeutic targets for immune-related and inflammatory diseases.

**FIGURE 1:**
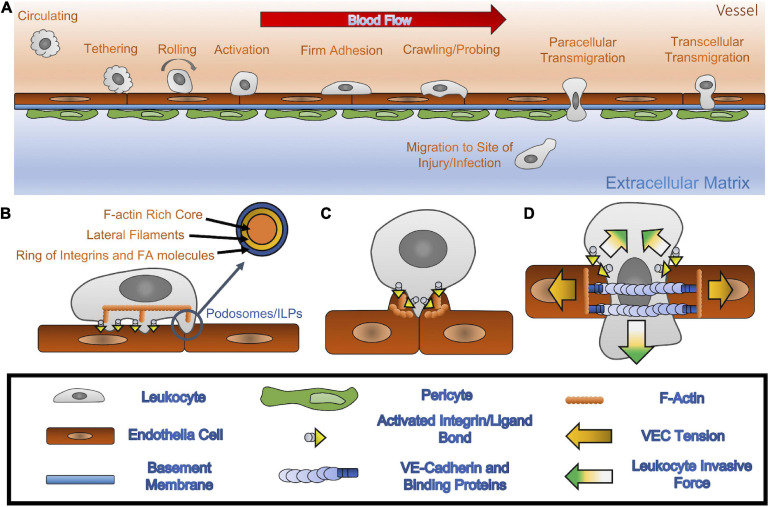
**(A)** Leukocyte extravasation: through the presence of inflamed VECs, circulating leukocytes localize themselves in the proximity of affected tissues. Once in range, leukocytes use carbohydrate ligands to tether themselves to VECs that express specific selectins. Once tethered, the leukocyte is then able to roll along the endothelium by creating and breaking bonds between the selectins and carbohydrate ligands. Upon the activation of integrins into a high affinity state, triggered by chemokines binding to leukocyte’s chemokine receptors, the leukocyte can transition into a firm adhesion state that stops the rolling and allows the leukocyte to spread out. The leukocyte then crawls and probes the vessel wall in search of VEC hotspots through which it is then able to transmigrate. This maneuver allows for leukocytes to breach the endothelium and basement membrane, thus permitting them to reach the affected tissue area. **(B)** Crawling/probing: leukocyte-VEC interactions, through high affinity integrins coupled with their respective CAMs, allow the leukocyte to migrate laterally, with the CAMs dictating the migration pattern of the leukocyte along the vascular wall. Furthermore, the leukocyte can convert focal adhesions to invadosome/podosomes like protrusive (ILP) structures, which are sensory organelles that they then utilize to search for TEM hotspots. **(C)** The transmigratory docking structure: once a hotspot is identified, a cluster of ICAM-1 creates a cup formation to hold on to the transmigrating leukocyte. This docking structure allows the leukocyte to transition from lateral migration to TEM. **(D)** TEM (paracellular): once in position at the sides of the VEC junction, leukocytes can increase VEC contractility, disrupting the local monolayer tension and creating strong downward pushing forces, which allow for a junctional gap to form and increase in size, and for invasion of the basement membrane. This widened gap allows for the leucocyte to push through the junction and break cellular bonds between VECs.

The scope and speed of the innate immune response are primarily dictated by transendothelial migration (TEM). The endothelium is formed by a monolayer of vascular endothelial cells (VECs) lining the vessel walls and functions as a physical barrier between the circulation and the underlying interstitial tissue. During TEM, leukocytes adhere to the VECs, transmigrate across the endothelium, and cross the vascular basement membrane to the extravascular space (Ley et al., 2007; Muller, 2014; Nourshargh and Alon, 2014; Vestweber, 2015; Figure 1). Pro-inflammatory stimuli such as TNF-α and IL-8 can activate both leukocytes and VECs to initiate TEM at the affected site (Middleton et al., 1997; Chandrasekharan et al., 2007). Circulating leukocytes bind to selectin molecules on the VECs via counter glycoprotein ligands, beginning a rolling and adhesion process (Kansas, 1996). Immobilized IL-8 chemokines on inflamed endothelial surfaces switch the leukocytes’ integrins LFA-1 and VLA-4 to high-affinity states, triggering the transition from rolling to firm adhesion and lateral migration, followed by direct TEM. The concomitance of high-affinity states in leukocyte integrins and increased expressions of ICAM-1 and VCAM-1 on inflamed VECs promotes cellular contractile forces, which regulate junctional integrity, endothelial permeability, and ultimately leukocyte trafficking (Cook-Mills and Deem, 2005; Stroka and Aranda-Espinoza, 2010). TEM occurs via one of two routes: at endothelial adherens junctions (paracellular migration) or through the VEC itself (transcellular migration). Although the factors governing route selection are not fully understood, both *in-vitro* and *in-vivo* experiments have demonstrated that the paracellular route is preferred, accounting for 90% of TEM events (Muller, 2003; Schulte et al., 2011; Woodfin et al., 2011). It remains unclear whether and how leukocytes probe the endothelium to find permissive sites for TEM and how leukocytes coordinate the force generation with VECs to facilitate their passage across the monolayer.

TEM involves several physical interaction cascades between leukocytes and VECs, characterized by the sequences of motions happening at the interfaces between the two cell types. Receptor-ligand interactions govern leukocyte TEM by modulating cellular functions, as mentioned above. For example, the activation of cell surface proteins triggers cytoskeletal rearrangements leading to increased cellular contractility and force transmission between the leukocytes, VECs, and the substrate. In this regard, TEM can be viewed as a biomechanically regulated process with contributions from both leukocytes and VECs. Recent advances in microfabrication, microscopy, and quantitative analysis allowed researchers to measure the mechanical forces involved in leukocyte-VEC interactions, contributing to delineating their roles in deciding the TEM route and driving cell motion. This review primarily focuses on two phases that play a determinant role in leukocyte trafficking: (1) adhesion and probing, and (2) direct TEM. Furthermore, we discuss current advances in force microscopy techniques for each phase and applications of force measurements in elucidating biomechanical mechanisms of leukocyte TEM. For additional background information on the biology of leukocyte TEM, we recommend previous reviews on this topic (Ley et al., 2007; Muller, 2014; Nourshargh and Alon, 2014; Vestweber, 2015).

## Biomechanics of Leukocyte Endothelial Adhesion and Probing

Almost immediately in response to pro-inflammatory signals, circulating leukocytes roll on the endothelial monolayer and then attach firmly to it (Figure 1A). Subsequently, they crawl over the endothelium using integrin-dependent adhesions. These interactions allow leukocytes and VECs to communicate by well-regulated surface receptors and their counter ligands on the opposing cell membrane. For example, the rolling step is mediated by rapid interaction between leukocyte selectin and P-selectin glycoprotein ligand-1 and endothelial P- and E-selectins (Alon et al., 1995; Lawrence et al., 1997; da Costa Martins et al., 2007; Hidalgo et al., 2007). Chemokine-induced integrin activation facilitates firm adhesion, spreading, crawling, and TEM by strengthening the leukocyte-VEC bond via leukocyte integrins (CD11/CD18, VLA-4) and their counter ligands, i.e., the adhesion molecules on VECs (e.g., ICAM-1, VCAM-1). This cascade of interactions has been characterized using specific blocking antibodies, pharmacological manipulations, and genetic perturbations to demonstrate each molecule’s role and downstream signaling effects *in vitro* and *in vivo* (Berlin et al., 1995; Huo et al., 2000; Singbartl et al., 2001; Chesnutt et al., 2006). This interaction cascade is also highly mechanically regulated. For example, during leukocyte rolling, the tensile forces on selectin catch-bonds have been shown to activate leukocyte integrins and facilitate leukocyte firm adhesion under shear stresses (Morikis et al., 2017).

In addition to regulating biochemical receptor-ligand interactions, leukocytes rely on mechanical forces to identify endothelial sites with decreased barrier function and to burrow through the endothelium. TEM does not occur with equal probability at all locations within the endothelium. Rather, it happens more often across the junctions between adjacent VECs than across the cytoplasm of single VECs. Moreover, it is observed more frequently at the confluence of three VECs (tricellular junctions) (Lampi et al., 2017) and between junctions loosened by inflammatory mediators (Schaefer and Hordijk, 2015). Following rolling and firm attachment to the endothelium, leukocytes spread out and initiate the protrusion and retraction of podosome-like structures that indent on endothelial membranes (Figure 1B). These structures are speculated to continually probe the underlying monolayer and play a decisive role in determining the leukocyte TEM route (Burns et al., 1997; Martinelli et al., 2014; Schaefer and Hordijk, 2015).

Podosomes are integrin-mediated adhesion structures observed in cells originating from myeloid tissue such as leukocytes and osteoclasts (Calle et al., 2006). These cells, especially leukocytes, migrate on comparatively soft substrates like endothelial or epithelial cells and their underlying interstitial tissues (Zen and Parkos, 2003; Sabri et al., 2006; Carman et al., 2007; Hidalgo and Frenette, 2007; Cougoule et al., 2010; Dehring et al., 2011). They develop their focal adhesions into specialized podosomes and invadosome/podosome-like protrusions (ILPs), all similar and highly specialized subcellular structures, when interacting with extracellular matrices and endothelial membranes, respectively (Martinelli et al., 2014). High-resolution microscopy revealed that the podosome supramolecular organization consists of a central F-actin core surrounded by a ring of integrins and focal adhesion molecules, including talin, vinculin, and paxillin (Pfaff and Jurdic, 2001; Vijayakumar et al., 2015; Foxall et al., 2019). The core and ring structures are interconnected by F-actin networks containing non-muscle myosin IIA (Pfaff and Jurdic, 2001; van den Dries et al., 2013, 2014). F-actin polymerization in the podosome core creates pushing forces, which are counterbalanced by the lateral pulling forces generated through actomyosin contractility in the cable-like structures connecting the core to adhesion sites (Labernadie et al., 2014). This actomyosin apparatus confers upon podosomes a highly dynamic behavior, including fast turnover times of a few minutes (Destaing et al., 2003; Evans et al., 2003) and the control over podosome growth, stiffness, and protrusive force generation (Labernadie et al., 2010, 2014; Bouissou et al., 2017).

These results have raised the fundamental question of how leukocytes utilize podosomes and ILPs to mechanosense their microenvironment. Podosomes generate forces via their actomyosin apparatus and sense their extracellular environments via the integrin-based ring substructures in association with mechanosensitive proteins, which activate downstream mechanotransduction pathways to control various cell functions. This process can be utilized to probe substrate topographies, trigger extracellular matrix degradation, and sense the stiffness of the surrounding matrix or underlying endothelium.

Studies on leukocyte adhesion to microfabricated substrates have found that leukocyte podosomes align themselves along substrate microgrooves (van den Dries et al., 2012). Because conforming to 3-D microgroove topographies alters leukocyte membrane curvature, this finding suggests that membrane curvature could play a critical role in regulating both the dynamics and spatial organization of podosomes. Given that the microtopographic features of tricellular VEC junctions can promote membrane curvature, specific subsets of protein and lipids associating with membrane curvatures (e.g., the BAR domain) might be involved in the podosome response to substrate topographies during the leukocyte TEM process.

Super-resolution microscopy has revealed that the F-actin podosome core is connected to a ventral F-actin module bound by vinculin and a dorsal module, crosslinked by myosin IIA and linked to other podosomes. Substrate stiffness influences the balance between these two modules allowing mesoscale podosome connections to collectively switch between the explorative, degradative behavior and the protrusive, non-degradative behavior (van den Dries et al., 2019). This stiffness sensing behavior is crucial for podosomes to explore spots compliant to protrusion. Moreover, clustered podosome force oscillations have been associated with expansion and retraction of the cell’s leading edge, demonstrating the exploratory role of podosomes during leukocyte migration (Kronenberg et al., 2017). The generation of vertical protrusive forces from cancer cell invadopodia has also been linked to cancer cell protease activity to degrade extracellular matrices (Aung et al., 2014; Dalaka et al., 2020). However, local disruption of integrin tensions in fibroblast podosomes had no effect on distal podosomes (Glazier et al., 2019), implying that collective podosome mechanosensing may be cell-type dependent and/or more complex than currently understood.

Given that integrins are a primary structural component of the podosome ring, chemokines play an essential role in podosome formation by promoting the high-affinity state of leukocyte integrins (Carman et al., 2007; Shulman et al., 2009). Immobilized or soluble chemokines bind to their receptors on leukocyte surfaces to regulate both actin polymerization at the core and integrin activation at the ring and promote the initiation of specific podosome architectures (Hoshino et al., 2013). However, there is no clear evidence showing any chemokine receptors exist on the podosome structures, and the detailed interplay between chemokine receptors and integrins will be needed for further investigations.

Vascular endothelial cells can modulate ILP activities by providing different ICAM-1 dependent ligand patterns (Andersen et al., 2016), which could influence how leukocytes select and migrate toward TEM hotspots. Conversely, ILPs have also been implicated in sensing VEC junctional integrity and cytoskeletal stiffness, and modulation of these factors has been shown to affect the TEM route (Martinelli et al., 2014). However, the precise nature of these biomechanical interactions is far from understood (Vestweber, 2015). Leukocyte ILPs are not just a sensory organelle and may, in fact, have additional functions. VECs regulate endogenous tension to maintain monolayer integrity and it is highly suspected that adhering leukocytes can alter this tensional balance (Yeh et al., 2018). For example, transcellular electron microscopy imaging suggests that ILPs may distort and bend underlying actin filaments inside of VECs by pushing directly on them (Martinelli et al., 2014).

In addition, ILPs display different characteristics from podosomes. In particular, ILPs on VECs have shorter lifetime than podosomes on extracellular matrices (seconds to mins vs. seconds to ten of mins) (Carman, 2009). Furthermore, leukocytes employ ILPs to probe the underlying VEC cytoskeleton and preferentially migrate toward compliant areas with low F-actin densities or loose junctions (Martinelli et al., 2014; Schaefer and Hordijk, 2015). Also, while leukocyte migration has been shown to vary with substrate compliance in substrates of uniform stiffness (Stroka and Aranda-Espinoza, 2009), there is a lack of data regarding leukocyte migration on substrates with stiffness gradients. Existing data on other cell types, however, suggest that integrin-mediated mechanosensing promotes durotaxis (i.e., migration toward stiffness gradient) rather than tenertaxis (Choquet et al., 1997; Lo et al., 2000; Vincent et al., 2013). Thus, there are still crucial outstanding questions regarding mechanosensing by trafficking leukocytes and the role of ILPs in this important cell function.

## Biomechanics of Direct Tem

After locating a hotspot on the endothelium, leukocytes shift from 2-D crawling to 3-D transmigration. Paracellular TEM is the most common route through the monolayer, mediated by the rapid disassembly of endothelial adherens junctions in response to an adherent leukocyte. The biomechanical interactions between leukocytes and VECs govern three crucial steps in this process. Specifically, mechanical forces contribute to opening endothelial gaps by destabilizing the junctions, help pull the leukocyte across the monolayer, and mediate the closure of the junctional gaps after TEM. This section discusses the currently recognized mechanisms and outlines open questions related to these three TEM steps.

### The Initiation of TEM: The Transmigratory Docking Structure

It has long been believed that VECs may play an active role in facilitating leukocyte TEM. VECs protrude microvilli-like projections perpendicular to the endothelium to form a specific “transmigratory docking structure” shaped like a cup, which can surround and hold an adherent leukocyte (Carman et al., 2003; Carman and Springer, 2004; Yang et al., 2005; Gerard et al., 2009; Teijeira et al., 2013). These structures are ICAM-1 or VCAM-1 enriched after actively binding to leukocyte integrin LFA-1 and VLA-4 (Barreiro et al., 2002; Carman and Springer, 2004; van Buul et al., 2007). Initially speculated to inhibit TEM (Carman et al., 2003), the docking structure is now understood to play an essential role in guiding leukocytes through the initial stages of transmigration (Carman and Springer, 2004). High-resolution time-lapse 3-D imaging has shown that ICAM-1 clusters appearing at docking structures during early TEM remain detectable surrounding the transmigrating leukocyte through the late stages of TEM (Carman and Springer, 2004). Disruption of these structures correlates with a reduction in TEM events (Carman and Springer, 2004). Of note, this imaging data revealed that ICAM-1 protrusions and docking structures align perpendicular to the endothelium (i.e., parallel to the direction of TEM). This spatial organization could help orient leukocyte integrins so that leukocytes can shift from 2-D lateral crawling and probing to an invasive 3-D migratory behavior.

The anchoring and embracing functions of VEC docking structures are regulated by mechanosensitive ICAM-1-triggered signaling, including recruitment of actin-binding proteins, an increase in F-actin assemblies, and activation of Rho-ROCK pathways, all of which result in increased actomyosin contractility (Yang et al., 2006; Heemskerk et al., 2014; Figure 1C). F-actin forms two types of assemblies with distinct functions in docking structures: (1) F-actin filaments extending ventrally from the apical side of endothelial membranes control VEC membrane protrusions while (2) F-actin rich cable-like structures confine transmigrating leukocytes at the basolateral side of VECs. In the early stages of TEM, vertically protruding F-actin filaments and VEC membrane fingers mediated by Myosin X activity secure adherent leukocytes (Franz et al., 2016; Kroon et al., 2018). The formation of these protrusions in inflamed VECs is regulated by the ICAM-1 cluster-mediated Cdc42-myosin-PAK4-F-actin signaling pathway, which generates mechanical forces to hold the leukocyte in place and subsequently pull it toward the VECs (Kroon et al., 2018). As TEM progresses, endothelial pores form to accommodate transmigrating leukocytes. Pore generation is regulated by the F-actin-rich cable-like structures (Heemskerk et al., 2016), which exert contractile forces against transmigrating leukocytes in order to maintain endothelial barrier functions throughout the entire TEM process and assist gap closure after it is complete (Mooren et al., 2014). Investigators employed inert beads coated with ICAM-1 antibodies to mimic adherent leukocytes, engage endothelial ICAM-1 clustering, and demonstrate the active role of VECs in TEM. These beads triggered a VEC process reminiscent of phagocytosis, in which VEC membrane extensions protruded to dock and embrace the beads (Carman et al., 2003; van Buul et al., 2010; Kroon et al., 2018). In addition, the functionalized beads were sufficient to induce strong localized VEC cellular traction forces (Liu et al., 2010; Yeh et al., 2018). The mechanical stresses created during docking structure formation and those observed during phagocytosis share common features, suggesting similarities between phagocytosis and leukocyte TEM (Vorselen et al., 2020a).

### The Crux of TEM: Junctional Gap Formation

Because leukocyte sizes can be more than 20 times greater than the size of endothelial cell-cell junctions (∼10 μm vs. ∼0.5 μm), transmigration must involve precise biomechanical coordination between leukocytes and VECs. VECs actively respond to the leukocyte’s presence by forming gaps to accommodate paracellular TEM. The activation of endothelial ICAM-1 through leukocyte binding can trigger a downstream signaling pathway that promotes cytosolic calcium-mediated myosin activity, resulting in increased endothelial contractility. The resulting increase in the tensile force supported by the F-actin cytoskeleton (i.e., endothelial tension, Figure 1D) is transmitted to VE-Cadherin, which connects F-actin to the VEC adherens junctional complex (Arif et al., 2021). This process causes the endothelial gap to enlarge for accommodating the transmigrating leukocyte (Shaw et al., 2001; Alcaide et al., 2008; Wee et al., 2009; Heemskerk et al., 2014). There is ample evidence that endothelial tension regulates paracellular TEM. Manipulating endothelial contractility by soluble inflammatory or anti-inflammatory agents such as thrombin and angiotensin I, biophysical cues such as stiff or soft subendothelial substrates, or by activating or inhibiting the RhoA GTPase all respectively increased or decreased the rates of leukocyte TEM (Hixenbaugh et al., 1997; Saito et al., 1998, 2002; Adamson et al., 1999; Carman et al., 2003; Yeh et al., 2018).

Apart from this VE-Cadherin-mediated junctional gap formation mechanism, the homophilic interaction between leukocyte PECAM-1 and VEC junctional PECAM-1 also plays a crucial role in leukocyte TEM by recruiting the lateral border recycling compartment (LBRC) to the site of TEM (Muller, 2003). LBRCs are networks of dynamic VEC vesicle-like membrane invaginations at cell-cell borders transported to TEM sites by kinesin motors along microtubules (Mamdouh et al., 2008). The primary molecule of LBRCs is endothelial PECAM-1, although they also contain other junctional molecules such as JAM-A and CD99 (Mamdouh et al., 2009). The LBRC surrounds the leukocyte, clears junctional VEC-Cadherin to open junctional gaps, and enlarges these gaps by contributing additional membrane material, all of which facilitates the transmigration process (Muller, 2014). Pharmacological perturbations inhibiting the formation or recruitment of LBRCs prevent TEM.

To complete the picture provided by the above studies, one must consider that leukocytes are mechanosensitive cells that exert forces during migration and invasion (Huse, 2017; Figure 1D). Recent 3-D traction force microscopy (TFM) studies have shown that leukocytes exert large burrowing stresses (∼1 KPa) to invade Matrigel substrates (Yeh et al., 2018). Simultaneous quantification of cell shape changes, position, and 3-D force exertion during leukocyte TEM revealed that burrowing vertical forces increase significantly during TEM events (Yeh et al., 2018). The tangential forces also become stronger and display a vector pattern directed inward toward VEC junctions, which lowers VEC monolayer tension (Yeh et al., 2018). In contrast, VEC monolayer tension raises when endothelial gaps form in response to mechanically inert anti-ICAM-1-coated beads. Moreover, gap formation mediated by these beads is significantly slower (∼120 min) than leukocyte TEM (∼10 min) (Yeh et al., 2018). Consistent with these findings, high-resolution correlative microscopy imaging of VEC cytoskeletal remodeling during TEM suggests that junctional gaps can be actively generated by leukocytes squeezing between adjacent VECs (Barzilai et al., 2017). In particular, the stiff leukocyte nucleus has been suggested to act as battering ram that displaces nearby VEC F-actin stress fibers to initiate and sustain junctional gaps (Barzilai et al., 2017).

Altogether, these studies show that junctional gap generation for TEM requires highly orchestrated biomechanical contributions from both leukocytes and VECs. Future studies are required to provide additional insight on exactly how these forces work together to promote leukocyte TEM.

### The Resolution of TEM: Junctional Gap

Preservation of endothelial barrier function requires that VEC junctional gaps be sealed once TEM concludes. The highly dynamic VEC F-actin cytoskeleton plays a crucial role in this process by continually polymerizing to form lamellipodial protrusions that make contact with neighboring VECs to signal gap closure (Mooren et al., 2014). Intact VECs exist under isometric tensions with contractile forces balanced by the cell-matrix and cell-cell adhesions (Charras and Yap, 2018). Gap formation during TEM is thought to disturb monolayer tension, triggering the formation of the F-actin lamellipodial structures that mediate gap closure (Phillipson et al., 2008; Martinelli et al., 2013). Concomitantly, Rho GEF Ect2- and LARG-activated RhoA signaling promotes actomyosin contractility in the F-actin-rich cable-like structures surrounding the leukocyte, similar to a purse string closure (Heemskerk et al., 2016). Inhibition of RhoA activity *in vitro* and *in vivo* did not affect the rate of leukocyte TEM events but caused leukocyte-induced vascular leaks. Moreover, a recent *in vivo* study found another Tie-2 receptor/Cdc42 GEF FGD5-stimulated mechanism responsible for preventing plasma leakage during leukocyte TEM. This study identified that platelets recruited to endothelial VWF activate the Tie-2 receptor by releasing Angiopoietin-1, reinforcing cable-like F-actin to close the endothelial pore (Braun et al., 2020). Further supporting the contribution of a purse string mechanism to maintaining barrier integrity during late TEM, the tangential traction stress patterns during gap closure are consistent with the presence of increased hoop tension (Yeh et al., 2018). Overall, these results indicate that F-actin-mediated signaling is essential for regulating gap closure, although there are not yet any direct measurements of endothelial monolayer tension during this process.

## Quantifying the Mechanics of Leukocyte Endothelial Crawling and Transmigration

The trans-well assay has been widely used to quantify leukocyte TEM for over two decades, unveiling several mechanisms crucial to this process. For example, leukocytes can actively respond to the exogeneous chemotactic gradient applied across the VEC monolayers such C5a or fMLP (Cooper et al., 1995). Without the application of exogeneous chemokines, the activation of VECs by TNF-α or IL-8 can induce expression and secretion of chemokines resulting in leukocyte integrin activation and the subsequent firm adhesion, crawling and transmigration (Adams and Lloyd, 1997). The trans-well device can easily create a chemotactic gradient via a micropore-based membrane separating the device’s upper and the lower chambers. However, this assay is not ideally suited to quantify the mechanics of TEM because it is not compatible with high magnification microscope objectives, customized substrates, and real-time imaging of most force microscopy methods.

**FIGURE 2:**
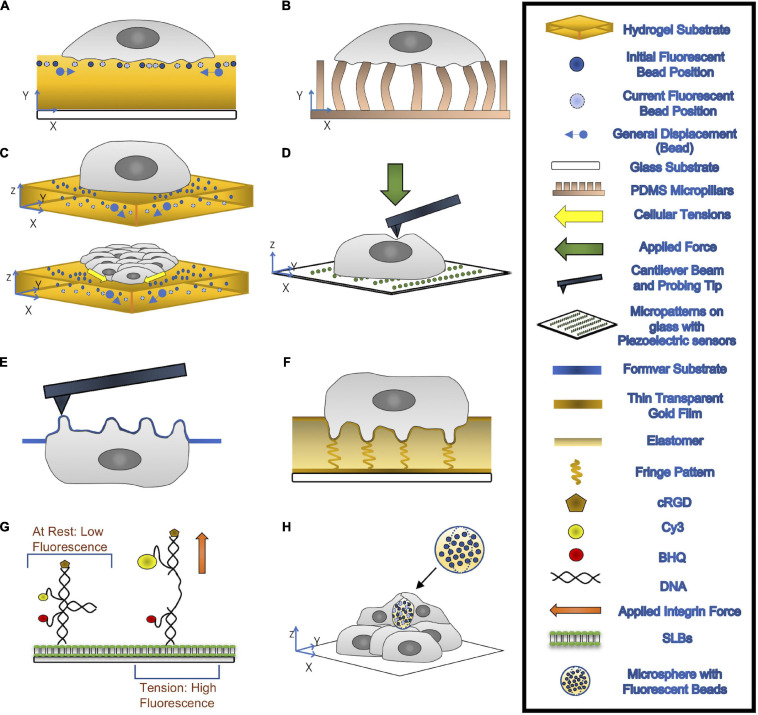
**(A,B)** 2-D TFM: cells are seeded on top of a hydrogel substrate containing fluorescent beads (**A**) or elastic micropillars **(B)**. Traction Forces can be measured through the imaging of the beads’ displacements or deflections of micropillars. **(C)** 3-D TFM (top) and 3-D MSM (bottom): 3-D TFM takes vertical direction displacement into account by comparing 3-D interrogate boxes between the deformed and undeformed state of substrates. 3-D MSM is derived from 3-D TFM and takes bending effects on cellular tensions into account. **(D)** AFM: A cantilever beam with a probing tip at one end applies a force onto the cell. **(E)** PFM: cells are seeded onto a formvar substrate, which is then stretched over a mesh grid. This formvar membrane is place upside, allowing for accurate topological information about the podosomes to be recorded through AFM. With the use of mathematical modeling, forces exerted by the podosomes can be calculated. **(F)** ERISM: thin transparent gold films are placed on top of and below an ultra-soft siloxane-based elastomer. The top gold film is protein coated to allow for cell adhesion. Local deformation caused by the cell to the elastomer form resonance fringes that are captured using established imaging modalities. **(G)** MT-FILM: surface functionalization of SLB with FRET-based DNA tension probe. When the integrin force applied is greater than *F*_*1/2*_, the rated force the probe can handle, the linker arms come together and open, causing the florescence of the probe increases. **(H)** microsphere-based TFM: Microspheres with fluorescent beads embedded are placed within multilayer of cells. After a few days, the cells exert compressive forces that deform the microspheres.

The most common current method to quantify cell-generated forces is TFM (Tan et al., 2003; Wang and Lin, 2007; Fu et al., 2010; Hall et al., 2013; Style et al., 2014; Polacheck and Chen, 2016; Figures 2A–C). This technique relies on measuring the cell-induced deformations in a continuous elastic substrate (e.g., a hydrogel) or an array of discrete elastic elements (e.g., microposts) of known mechanical properties. Using these data as inputs, a mathematical inverse problem is solved to recover traction forces (Style et al., 2014; Polacheck and Chen, 2016). TFM experiments have revealed that, similar to other cell types (Hur et al., 2009; Legant et al., 2013; Alvarez-Gonzalez et al., 2015), neutrophils and macrophages in an adherent state exert pulling forces around the cell edge and an unstructured bulk pushing force under the main cell body (Kronenberg et al., 2017; Yeh et al., 2018). The observation of subtle variations in the bulk pushing force has been attributed to the force from podosomes (Kronenberg et al., 2017). The joint requirements of fine spatial resolution and high temporal sensitivity to measure such minute forces make it challenging to quantify podosome exerted forces by TFM. Thus, specialized methods based on atomic force microscopy (AFM) and molecular sensors have been developed for this application. Finally, VEC contractility contributes to building tissue-level mechanical tension in monolayers, which is modulated in response to external stimuli such as flow shear stresses or the presence of an adhering leukocyte (Hur et al., 2012; Yeh et al., 2018). Motivated by reports showing that monolayer tension is known to regulate junctional integrity and endothelial barrier function (Tornavaca et al., 2015; Andresen Eguiluz et al., 2017), monolayer stress microscopy (MSM) techniques have been developed to measure this tension. Below, we discuss techniques developed to quantify the mechanics of leukocyte TEM. These techniques are illustrated in Figure 2 and their specific applications, strengths, and limitations are summarized in Table 1.

**TABLE 1:** Summary of cellular force measurement techniques.

	**Resolution (x/y/z/F/t)**	**Cell substrate**	**System requirements**	**Post-processing**	**Advantages**	**Disadvantages**	**References**
Traction force microscopy (TFM) Figures 2A–C	Depends mostly on substrate properties and microscopy setup	Hydrogel, PDMS, elastomers, fibrillar matrices (e.g., collagen); Arrays of elastic micropillars	Standard fluorescent microscope; Confocal microscopy for out of plane bead tracking and 3-D tractions	Single particle tracking, correlation tracking, and/or particle image velocimetry; Theoretical/computational solid mechanics analysis	Cell substrate can be physiologically realistic (except micropillar arrays); Image based – highly versatile; Simple experimental setup and high throughput; Can be extended to provide collective cellular force measurements (e.g., monolayer stress microscopy)	Requires zero force state (except micropillar arrays) and calibrating substrate elastic properties; Limited sensitivity to vertical forces; Fluorescence microscopy over long periods can cause phototoxic effects	Tan et al., 2003; Wang and Lin, 2007; Fu et al., 2010; del Alamo et al., 2013; Hall et al., 2013; Style et al., 2014; Polacheck and Chen, 2016; Serrano et al., 2019
Atomic force microscopy (AFM) Figure 2D	Resolution depends on the imaging force and probe geometries; Lateral resolution 1–1.5 nm; Vertical resolution 0.1 nm; Force resolution 100 pN	Mica, glass, or glass slides modified with Silane to enhance cell adhesions	Piezoelectric scanner for mounting samples; Proper probes attached to pliable silicon or silicon nitride cantilever; Laser beam/photodiode setup for measuring cantilever deflection	Cantilever deflection as a function of vertical displacements; Conversion a force-versus-separation distance curve	Probes for molecular interactions, physiochemical properties, surface stiffnesses, and macromolecular elasticities	Requires careful sample preparation and data collection; Requires physical contact between the AFM probe and the sample – cannot probe basal structures (e.g., podosomes tips) Localizing specific cell structures (e.g., podosomes) by AFM alone is challenging	Labernadie et al., 2010
Protrusion force microscopy (PFM) Figure 2E	The same as AFM; Vertical resolution 10 nm; Line rate on order of 1 Hz; Force resolution to the order of nN	Compliant formvar membranes	AFM system and fluorescence microscopy	The same as AFM, plus mathematical model to infer podosomes forces from formvar membrane deformation	Measures protrusive forces applied perpendicularly to the substrate at a single podosome level; High spatiotemporal resolution	The same as AFM, except for localizing podosomes; Narrow range of applications.	Labernadie et al., 2014; Bouissou et al., 2017
Elastic resonator interference stress microscopy (ERISM)*Figure 2F*	Displacement resolution 2nm (limited by surface); Temporal resolution <0.5 s; Lateral resolution ∼1.6 μm;	Elastic optical micro-cavity comprized of a layer of ultra-soft siloxane-based elastomer sandwiched between semi-transparent gold layers	Conventional wide-field phase contrast or fluorescent microscopy with a tunable light source capable of providing monochromatic illumination	Each light fringe ∼ 200 nm = ≥ count fringes to determine size of deformations; Conversion of forces by utilizing substrate mechanical properties	Unlike many TFM methods, no zero-force state required; No phototoxic effects; Versatile, and compatible with other microscopy methods; Excellent vertical and lateral force sensitivities	Experimental setup and fabrication of ERISM cavities are relatively involved; 2D soft substrate may not be physiologically realistic for some applications	Kronenberg et al., 2017; Liehm et al., 2018
Molecular tension-fluorescence lifetime imaging microscopy (MT-FLIM) Figure 2G	Force threshold F_1/2_ is a measure of the applied force at which 50% of probes are open, estimating applied force ranges; Force resolution to the order of pN exerted by individual integrins	Supported lipid bilayer (SLB) – phospholipid membranes; Confined in the Z-direction but are laterally fluid	Inverted microscopes with perfect focus capabilities and appropriate lasers for excitation; Specific software required;	Matlab Bioformats Toolbox and semiautomated custom scripts; FIJI plugins, including MultiKymograph and TrackMate	Highly specific observations of integrin behavior and force generation as it relates to podosome formation and mechanosensing	Highly technical in the development and implementation of molecular tension probes, and in microscopy set-up Misses forces transmitted via non-specific interactions	Wang and Ha, 2013; Blakely et al., 2014; Zhang et al., 2014; Brockman et al., 2018; Glazier et al., 2019
Microsphere-based traction force microscopy Figure 2H	Force resolution to the order of nN	Hydrogel-based microspheres; Fabricated by water-oil emulsions	Standard fluorescent microscope; Confocal microscopy for tracking 3-D shape deformations	Comparison between deformed and undeformed states from 3-D shape reconstructions	Suitable for studying forces in environments with complex mechanical properties, where TFM and ESRIM would be challenging	Intensive image processing requirements Resolution is limited by spatial distribution of microspheres in sample	Girardo et al., 2018; Mohagheghian et al., 2018; Kaytanli et al., 2020; Vorselen et al., 2020b

### Quantifying Subcellular Forces

Early efforts to assess the dynamics and physical properties of podosomes combined the application of AFM, micropatterned substrates, and correlative fluorescent microscopy (Labernadie et al., 2010; Figure 2D). Macrophages were plated on a glass coverslip patterned with arrays of fibrinogen squares, encouraging podosome formation in the protein spots in order to restrict the size of the analysis field. AFM, in which a cantilever applies a known force to the substrate in order to determine its stiffness, was then used to identify the location of membrane bumps corresponding to fluorescently labeled F-actin rich structures (Henderson et al., 1992; Lu et al., 2008). Time series of AFM topological images enabled accurate measurement of podosome height (mean 578 ± 209 nm) while force-distance mapping provided a wealth of information on podosome stiffness. Not only was the mean Young’s modulus of podosomes found to be approximately five times higher than surrounding regions, but rapidly acquired force-distance curves reproducibly demonstrated periodic oscillations in podosome stiffness (Labernadie et al., 2010). This targeted application of AFM permitted a refined analysis of podosome structure and function. However, it also highlighted several key limitations of standard AFM to quantify the biomechanics of podosomes. Specifically, the impossibility of probing the basal tip of the podosome which is in contact with the substrate, the difficulty of localizing podosomes from profiles of apical cell height, and, most notably, the impossibility of measuring podosome protrusive forces.

In order to address these limitations, protrusion force microscopy (PFM) was developed as an extension of standard AFM to measure indentations made by cells seeded on a specially fabricated, compliant formvar substrate (Labernadie et al., 2014; Figure 2E). The formvar membrane, stretched over a square mesh grid, was seeded with cells before being mounted upside down in the microscope so that AFM could be performed directly on the membrane bumps caused by podosomes. The force generated by each podosome was calculated by fitting the height and radius of each protrusion to a mathematical model of a clamped membrane subject to a point force (Labernadie et al., 2014). By increasing the thickness of the formvar sheet and thus the substrate stiffness sensed by cells, PFM results demonstrated that leukocyte podosomes increase their protrusive forces in response to stiffer substrates, suggesting that podosomes have a mechanosensing function (Labernadie et al., 2014). Periodic oscillations in podosome protrusive forces concomitant with the aforementioned periodic stiffness oscillations were uncovered using time-lapse PFM, which functions by keeping the AFM cantilever tip at a constant force in contact with the top of a protrusion as it moves in real-time (Labernadie et al., 2014). The similarities between periods of podosome protrusive forces (40 ± 14s), core stiffness (37 ± 20s) as measured by PFM, and F-actin intensities at the podosome core (44 ± 11s) as measured by total internal reflection fluorescent imaging indicate a correlation between the generation of oscillating protrusion forces and the stiffness and F-actin content of the podosome core. This correlation was validated by pharmacological perturbations, demonstrating that both F-actin polymerization and actomyosin contractility regulate periodic protrusion forces.

While PFM provides invaluable information about the biomechanics of podosomes, it is a highly specialized technique. For instance, it is not straightforward to integrate PFM measurements of protrusive forces with long-term, whole-cell measurements of tangential traction stresses and motility. The emergence of elastic resonator interference stress microscopy (ERISM, Figure 2F) now allows for quantifying whole-scale lateral and protrusive cell-substrate forces, with enough resolution to detect the minute forces exerted by podosomes and over extended periods of time. ERISM implements interferometric detection of cell-induced deformations in an elastic microcavity, allowing for high sensitivity to weak forces in a non-destructive manner such that cells can be retained on the substrate after imaging for subsequent measurements and assays (Kronenberg et al., 2017; Liehm et al., 2018). ERISM measurements could in principle be analyzed to recover collective cellular stresses such as endothelial monolayer tension. Thus, although it cannot currently be applied to 3-D physiologically relevant environments like, (e.g., Matrigel or collagen matrices), ERISM constitutes a promising technique to quantify the biomechanics of leukocyte TEM.

Pursuant to these developments, specialized microscopic methods were developed to investigate the contributions of specific proteins to podosome force generation. Specifically, DNA-based subcellular tools were developed to explore the role of integrin tensile forces in podosome formation and illustrate the mechanical link between integrin tension at the podosome ring and actin protrusion at the podosome core (Glazier et al., 2019; Figure 2G). Molecular Tension-Fluorescence Lifetime Imaging Microscopy (MT-FLIM) allows for precise, piconewton (pN) resolution measurement of integrin tensile forces. MT-FLIM relies on the surface functionalization of a laterally fluid, self-assembling phospholipid membrane on glass (supported lipid bilayer, or SLB) with FRET-based DNA tension probes (Wang and Ha, 2013; Blakely et al., 2014; Zhang et al., 2014; Brockman et al., 2018; Glazier et al., 2019). The probes consist of a binary DNA hairpin with an internal loop structure and two linker arms hybridized to include (1) a Cy3/BHQ FRET pair, which is brought into proximity when a force greater than the tunable F_1/2_ threshold is applied to the probe, opening the hairpin and (2) cyclic Arg-Gly-Asp-*D*-Phe-Lys (cRGD), which is localized on the upper arm and whose depletion is a proxy for podosome protrusive forces as measured by percentage decrease in fluorescence. Application of a force greater than or equal to F_1/2_ will open the probe, increasing both fluorescence intensity and lifetime (Glazier et al., 2019). Using tunable F_1/2_ DNA probes, MT-FLIM enabled the identification of a narrow range of integrin mediated forces and used time-course imaging to establish a picture of the spatiotemporal evolution of podosome force generations at a subcellular level.

### Quantifying Single Cell Forces

Two-dimensional (2-D) TFM, which measures the lateral traction forces parallel to the surface of cell attachment, was first applied by Dembo and Wang to fibroblasts migrating on flat substrates (Dembo and Wang, 1999). This technique has been widely used to quantify the biomechanics of leukocyte adhesion and crawling on substrates of varying stiffness of 0.05–8 kPa, providing traction stress maps with a lateral resolution of 1–5 μm (Rabodzey et al., 2008; Liu et al., 2010; Yeh et al., 2018). The experimental assay used in this type of experiments has been refined over the years and consists of a protein-coated gel (e.g., polyacrylamide) containing fluorescent microspheres near its surface (Figure 2A). Substrate deformations are quantified from the movement of the fluorescent beads by image correlation techniques, using reference images obtained after treating the cells (e.g., by trypsin) to detach from the substrate or after the cells move away from the region of interest. The partial differential equation of elastic equilibrium for the substrate (i.e., the elastostatic equation) can be solved to determine the traction stresses from the measured deformations using a variety of inversion and regularization procedures (Schwarz et al., 2002; Sabass et al., 2008). Notably, the computationally efficient Fourier analysis of the elastostatic equation proposed by Butler et al. (2002) makes it possible to calculate traction stresses from raw microscopy images virtually in real time.

In micropost-based TFM, protein-coated arrays of microscopic pillars made from the deformable elastomer polydimethylsiloxane (PDMS) serve as a substrate (Figure 2B). Cells attached to these arrays induce pillar deflections that can be converted into force vectors using the known height, width, and material properties of the pillars (Tan et al., 2003; Fu et al., 2010; Polacheck and Chen, 2016). In principle, micropost-based TFM does not require a reference image since the undeflected positions of the pillars are known theoretically (Lemmon et al., 2009), which is advantageous. On the other hand, microfabrication and imaging constraints limit the spatial resolution of this technique (Amato et al., 2012). Furthermore, the highly particular substrate topography created by the micropost arrays differs from physiologically relevant scenarios and can affect cell adhesion. Pioneering micropost-TFM studies of leukocyte endothelial crawling showed that VECs exert increased tangential forces in response to a firmly adherent leukocyte, uncovering that biomechanical interactions between VECs and adherent leukocytes and play a role in TEM (Rabodzey et al., 2008; Liu et al., 2010).

The 2-D TFM is relatively straightforward on standard fluorescent microscopes, has excellent resolution, and can be adapted to a wide variety of applications (Style et al., 2014; Polacheck and Chen, 2016). However, leukocyte TEM is an inherently 3-D process involving significant forces in the vertical direction of invasion. TFM measurement of these vertical forces requires more involved imaging setups and careful postprocessing to balance resolution with phototoxic effects. Motivated by the fact that cells generate both lateral and vertical traction forces while adhering to and migrating over planar substrates (Hur et al., 2009; Maskarinec et al., 2009), TFM methods to measure these 3-D forces have been developed (Hur et al., 2009; Maskarinec et al., 2009). These techniques are loosely referred to as 2.5-D TFM because they provide 3-D traction forces in the 2-D plane of cell attachment, differentiating them from volumetric TFM experiments where cells are fully embedded inside 3-D matrices (Legant et al., 2010). In 2.5-D TFM experiments the substrate is the same as in 2-D TFM, but confocal imaging is required to record 3-D substrate deformations. To minimize the phototoxicity generated by laser radiation when acquiring a z-stack of confocal images, del Alamo et al. developed a methodology that inputs the 3-D deformation at the top plane of the substrate into the solution of the 3-D elastostatic equation (del Alamo et al., 2013), thus requiring only ∼10-slice z-stacks (or approximately 10 μm in depth). By adding deformation data from additional planes, this methodology can be extended to substrates of unknown mechanical properties or used to detect substrate degradation (Aung et al., 2014; Alvarez-Gonzalez et al., 2017). Overall, 2.5-D TFM constitutes a powerful tool to study the biomechanics of TEM and delineate the distinct roles of VEC and leukocyte forces in coordinating this process.

### Quantifying Tissue-Level Cell Forces

In order to maintain homeostatic barrier function, VECs must regulate their monolayer tension to balance the biomechanical stability of cell-cell junctions and cell-substrate adhesions. This balance prevents cell adhesion forces from tearing the endothelium apart or detaching it from the substrate (Charras and Yap, 2018). During inflammatory responses, both the magnitude and fluctuations of VEC monolayer tension tend to increase, leading to inherently unstable junctions (Yeh et al., 2018). Measurements of endothelial monolayer tensions over time suggest that the rate of leukocyte TEM correlates with tension fluctuations, which can be actively induced by leukocytes at TEM sites (Yeh et al., 2018). Recent mathematical models also support the idea that monolayer tension fluctuations play a crucial role in monolayer integrity and leukocyte TEM (Escribano et al., 2019). However, joint quantitative measurements of endothelial traction forces, monolayer tensions, and the forces exerted by leukocytes on the endothelium are still scarce.

In comparison to AFM or TFM, the development of experimental, image analysis, and computational tools to quantify collective cellular forces has been recent. A salient technique is MSM, an extension of TFM that quantifies the collective distribution of intracellular stresses in thin confluent cell layers (Trepat et al., 2009). Of note, MSM can measure monolayer tension, which is the tensile intracellular stress. Most MSM methods calculate intracellular stresses in the monolayer from 2-D measurements of in-plane traction stresses by applying Newton’s third law in differential (Trepat et al., 2009) or integral (Hur et al., 2012) form after averaging across monolayer thickness. The differential formulation provides significantly better lateral spatial resolution than the integral one, although it relies on a number of simplifying assumptions such as linearly elastic material behavior, constant elastic moduli, and known Poisson ratio. For the most part, these assumptions do not seem to severely affect the recovered intracellular stresses (Trepat et al., 2009; Tambe et al., 2013). Furthermore, they can be relaxed using particle dynamics simulations (Zimmermann et al., 2014) or Bayesian inference analyses (Nier et al., 2016). However, these 2-D approaches do not consider that cell monolayers respond to not only in-plane tangential stresses, but also out-of-plane stresses that induce monolayer bending. Confluent VECs adhering to soft substrates can generate strong out-of-plane traction stresses that bend the monolayer, particularly near the monolayer edges (Serrano et al., 2019). The invasive forces exerted by leukocytes during TEM also cause monolayer bending, leading to significant perturbations in intracellular tension (Yeh et al., 2018). To overcome the limitations of 2-D MSM, Serrano et al. recently developed a new MSM method (Serrano et al., 2019) that uses 2.5-D TFM measurements to calculate the contributions of lateral and bending deformations to monolayer tension (Figure 2C).

An inherent difficulty in quantifying the biomechanics of TEM is to tease out the forces exerted by the leukocytes from those exerted by the VECs. To this end, ICAM-1 antibody-coated polystyrene beads mimicking firmly adherent leukocytes have been used in combination with TFM and MSM methods (Liu et al., 2010; Yeh et al., 2018; Serrano et al., 2019). Given that the microbeads are mechanically inert, these experiments provide useful information about how VECs regulate monolayer tension during TEM. However, polystyrene beads are rigid, which makes it impossible to quantify the forces that VECs exert on the beads, and the recent development of methods to quantify mechanical forces *in vivo* via deformable hydrogel microspheres could overcome this limitation. Inspired by the seminal use of functionalized oil droplets to measure anisotropic stresses within 3-D cell aggregates (Campas et al., 2014), emerging microfabrication methods can now produce deformable hydrogel-based spherical force sensors, with sizes ranging from a few μm up to hundreds of microns (Girardo et al., 2018; Mohagheghian et al., 2018; Kaytanli et al., 2020; Vorselen et al., 2020b; Figure 2H). These elastic microspheres can be employed to study cellular forces induced by specific ligand-receptor interactions, known as microsphere-based TFM. Comparisons between the deformed and the stress-free state of microspheres allows force measurements to be performed using analysis methods similar to those employed in TFM. These techniques are anticipated to generate novel quantitative insights about the mechanical progression of VEC docking structure formation during the initial TEM process.

Finally, it is important to note that, although shear stress is an important regulator in inducing leukocyte TEM (Cinamon et al., 2001), most existing force microscopy studies of leukocyte TEM have not considered shear flow conditions so far. While it requires a more complicated experimental setting, it is not unfeasible to consider shear flow in force microscopy assays and, in fact, there are established TFM and FRET imaging assays that include shear flow (Hur et al., 2012; Perrault et al., 2015; Heemskerk et al., 2016). Future efforts shall exploit these tools to study how shear affects the mechanics of leukocyte TEM by directly measure the forces involved in the process.

## Concluding Remarks

Leukocyte recruitment is a hallmark of all acute and chronic inflammatory disorders. Understanding leukocyte TEM to inflammation sites could help identify therapeutic targets to boost immune defense and minimize inflammatory tissue damage. Currently, medical treatments of chronic inflammation employ general immune suppressors, which have numerous adverse side effects. This lack of success is attributed to the complexity of and the multitude of redundancies and interdependencies in the molecular pathways involved. Although many molecules, some of which are discussed above, have been implicated in TEM, their specific roles remain elusive. In particular, we still know little about how VECs and leukocytes orchestrate pathfinding and localized endothelial barrier modulation. These processes can depend on direct receptor-ligand biomechanical interactions and more intricate, collective mechanosensitive pathways. Their understanding requires advanced experimental techniques to detect subcellular, single-cell, and tissue-level deformations during leukocyte TEM, along with the corresponding development of computational models to analyze data streams of increasing complexity and size.

During the past ten years, the progress in the development of soft materials have brought *in vitro* assays close to reproducing physiological microenvironments under controlled conditions. In parallel, advances in 3-D imaging and force quantification have opened a window to data of unprecedented richness and quality. Indeed, these methodologies have significantly extended our current understanding of leukocyte trafficking. The current technological frontier is methods to allow investigation of these biomechanical events *in vivo*. Of note, emerging microscopy techniques such as two-photon, light sheet, and super-resolution microscopy will most likely play a transformative role in bridging the gap going from *in vitro* conditions to more realistic animal models. Heavy interdisciplinary efforts involving engineers, physicists, and mathematicians will certainly be required to overcome these formidable challenges.

## Author Contributions

AS, EC-H, SC, JÁ, JL, and Y-TY wrote the manuscript. AS and OC composed the figures and table. All authors contributed to the article and approved the submitted version.

## Conflict of Interest

The authors declare that the research was conducted in the absence of any commercial or financial relationships that could be construed as a potential conflict of interest.
